# Breaking barriers in the prevention of adolescent pregnancies for in-school children in Kirehe district (Rwanda): a mixed-method study for the development of a peer education program on sexual and reproductive health

**DOI:** 10.1186/s12978-020-00986-9

**Published:** 2020-09-07

**Authors:** Aimable Nkurunziza, Nadja Van Endert, Justine Bagirisano, Jean Bosco Hitayezu, Sylvie Dewaele, Olive Tengera, Goele Jans

**Affiliations:** 1 School of Nursing and Midwifery, College of Medicine and Health Sciences, University of Rwanda, Kigali, Rwanda; 2 Arthur Labatt School of Nursing, Faculty of Health Sciences, University of Western Ontario, London, Canada; 3 University Colleges Leuven-Limburg, Research & Expertise, Resilient People, Wetenschapspark 21, 3590 Diepenbeek, Belgium; 4 University Colleges Leuven-Limburg, Department of Health, Midwifery program, Campus LiZa, Schiepse Bos 3, 3600 Genk, Belgium

**Keywords:** Unintended pregnancies, Peer education, Adolescents, Sexual and reproductive health

## Abstract

**Background:**

Despite a variety of mainly school-driven prevention strategies, the number of adolescent pregnancies in Rwanda is worryingly high and is even expected to increase. The aim of this study is to empower Kirehe secondary school students aged 15–19 years old in sexual and reproductive health (SRH) by developing a peer education program.

**Methods:**

A combination of quantitative and qualitative research will be used. A pre- and post-survey will examine adolescents’ knowledge and attitudes regarding SRH. In addition, six focus group interviews will explore these knowledge, attitudes but also SRH needs more in depth. Based on the obtained information, and after retrieving experts’ input, a peer education program is being developed in which Midwifery students obtain training in SRH and educational skills (= first train-the-trainer module). In turn, these students will educate and train a selected group of secondary school students (= second train the trainer module). Finally, these trained in-school students can act as reliable peers for other in-school students in the context of SRH.

**Discussion:**

The project will contribute to 1) more independent and thoughtful decisions in contraception and sexual behavior, and consequently less adolescent pregnancies, and 2) the reinforcement of the Rwandan Midwifery education.

**Trial registration:**

University of Rwanda, College of Medicine and Health Sciences, Institutional Review Board, Approval No 158/CMHS IRB/2019.

## Plain English summary

Despite a variety of mainly school-driven prevention strategies, the number of adolescent pregnancies in Rwanda remains high. The aim of this VLIR-UOS project, which is a North-south collaboration between University Colleges Leuven-Limburg and the University Of Rwanda, is to develop a peer education program (PEP) in the context of SRH for Kirehe in-school adolescents aged 15 to 19 years old. In a first phase, adolescents’ needs, knowledge and attitudes regarding SRH were examined. A cross-sectional survey was conducted in three secondary schools in Kirehe district. Based on the obtained information from the survey, six additional focus group interviews, and after retrieving experts’ input, the PEP is being developed in which University of Rwanda Midwifery students obtain training in SRH and educational skills (= first train-the-trainer module). In turn, these students will educate and train secondary in-school students (= second train the trainer module). Finally, these trained in-school students can act as reliable SRH peers for other in-school adolescents. Midwifery students play a key role in training of peers, who in turn could reinforce others in the context of contraception, AIDS and STDs. A PEP on SRH can be an effective complementary strategy for the empowerment of Kirehe in-school adolescents, contributing to more independent and thoughtful SRH decisions, and consequently to less adolescent pregnancies.

## Background

Adolescent pregnancies are an important global problem. Yearly, approximately 21 million 15 to 19-years old girls become pregnant in developing regions, and approximately 16 million of them actually give birth [1, 2]. It is well demonstrated that adolescent pregnancies are associated with higher risks of eclampsia, puerperal endometritis, low birth weight and preterm delivery compared to pregnancies in women aged 20–24 years old. Complications of pregnancy and childbirth are even the second leading cause of death among 15–19-year-old women. In addition, there are economic and social consequences affecting the adolescents, as well as their parents and the community. Stigma, rejection, more violence within marriage, lower level of education, less employment opportunities and maintained cycles of poverty, are only few concerns being addressed [3].

Despite the overall progress, projections indicate that the global number of adolescent pregnancies will increase by 2030. Reason for this is the continued growth of the population of adolescents. The Republic of Rwanda is ranked fifth of the countries with the highest percentage increase of the adolescent population, with a proportional increase in adolescent girls of 75% [4].

Despite an overall improvement in most aspects of health in Rwanda, adolescent pregnancies are increasing from 6.3% in 2010 up to 7.3% (*n* = 818), in regard to the total number of pregnancies in 2015. The highest percentage of adolescent pregnancy is observed in Huye District (14.2%) [5]. However, when examining more precisely the distribution of these data, it is alarming that the problem mainly exists in adolescents attending secondary school. In this context, also Kirehe district draws the attention. Approximately 85% of all adolescent pregnancies is observed in adolescents attending school, and only a small proportion in adolescents who do not enjoy any form of official education [6]. This is somehow surprising, since adolescent pregnancies are more likely to occur in communities with low education and employment opportunities [7]. On the other hand, Rwandan public press published articles on pregnancy scandals in Rwandan schools (e.g. http://www.newtimes.co.rw/section/read/62481) caused by adults in powerful positions taking sexual advantage of young adolescent girls. This clearly illustrates the complex given of adolescent pregnancies, not solely being an issue of adolescent SRH education, but resulting from a complex synergy between socio-cultural, economic and personal factors [7].

### Need for more research?

The Rwandan government has strongly invested in SRH by mainly a variety of school-based programs, such as the further development of Biology and Health Sciences classes from senior three to six classes. In addition, the Rwanda Medical Center supervises a SRH teaching program for in- and out-school adolescents which is supported by Imbuto Foundation and UNFPA. To date, these programs seem to be less effective than expected [8, 9]. School-based programs still face the problem of discomfort and taboo with SRH in the context of teachers and students. Consequently, adolescents seek information on SRH from their peers, but this poses a first problem with the reliability of the exchanged information, as research has shown that peers are often not correctly informed on SRH-topics [5]. Second, peers are not always fully accessible to all adolescents, so that the needs of some remain unmet. In summary, Rwandan secondary students remain to have low level of knowledge regarding SRH and, on top, risky sexual decision making is influenced by important factors such as social influence, social context and interaction [10].

### Aim and objectives

The objective of this study is to contribute to the reduction of adolescent pregnancies by obtaining empowered Kirehe in-school adolescents who make more and better use of means to prevent adolescent pregnancy. We aim to achieve this objective by:

1. Strengthening the Midwifery programs in SRH education in order to obtain well equipped en trained Midwifery and Nursing students;
2. Educating and training a selected group of secondary school students who can connect to other secondary school students for accurate exchange in SRH knowledge and skills.

The final outcome of this study is the development of a scientifically and practically based peer education program for Kirehe in-school adolescents in the context of SRH.

## Methods/design

### Vlir-uos south project

This study can be described within a three-phased participative and reflective process as part of structural partnership of 8 years between University of Rwanda (UR) and University Colleges Leuven-Limburg (UCLL). A partnership with various official and structured contacts and exchanges is built with the aim of reinforcing local capacities on education, research and outreaching services. This partnership is officially recorded in a Memorandum of Understanding between both institutions, signed in December 2017.

The study is product of a multi-departmental training with UR and UCLL members and is translated into a VLIR-UOS project. VLIR-UOS is an official platform with the aim of supporting partnerships between universities and university colleges in Flanders and the South. It particularly supports innovative responses to global and local challenges [11]. The country program 2017–2021 for Rwanda targets the following five main themes: 1) food security, 2) natural resources management, 3) Environment, 4) Human rights and government, and 5) Health [12]. The current project is located in the Health section with project number RW2019SIN259B141. UCLL acts as the coordinating partner, UR as the local south partner.

### Study design, setting and population

**Fig. 1:**
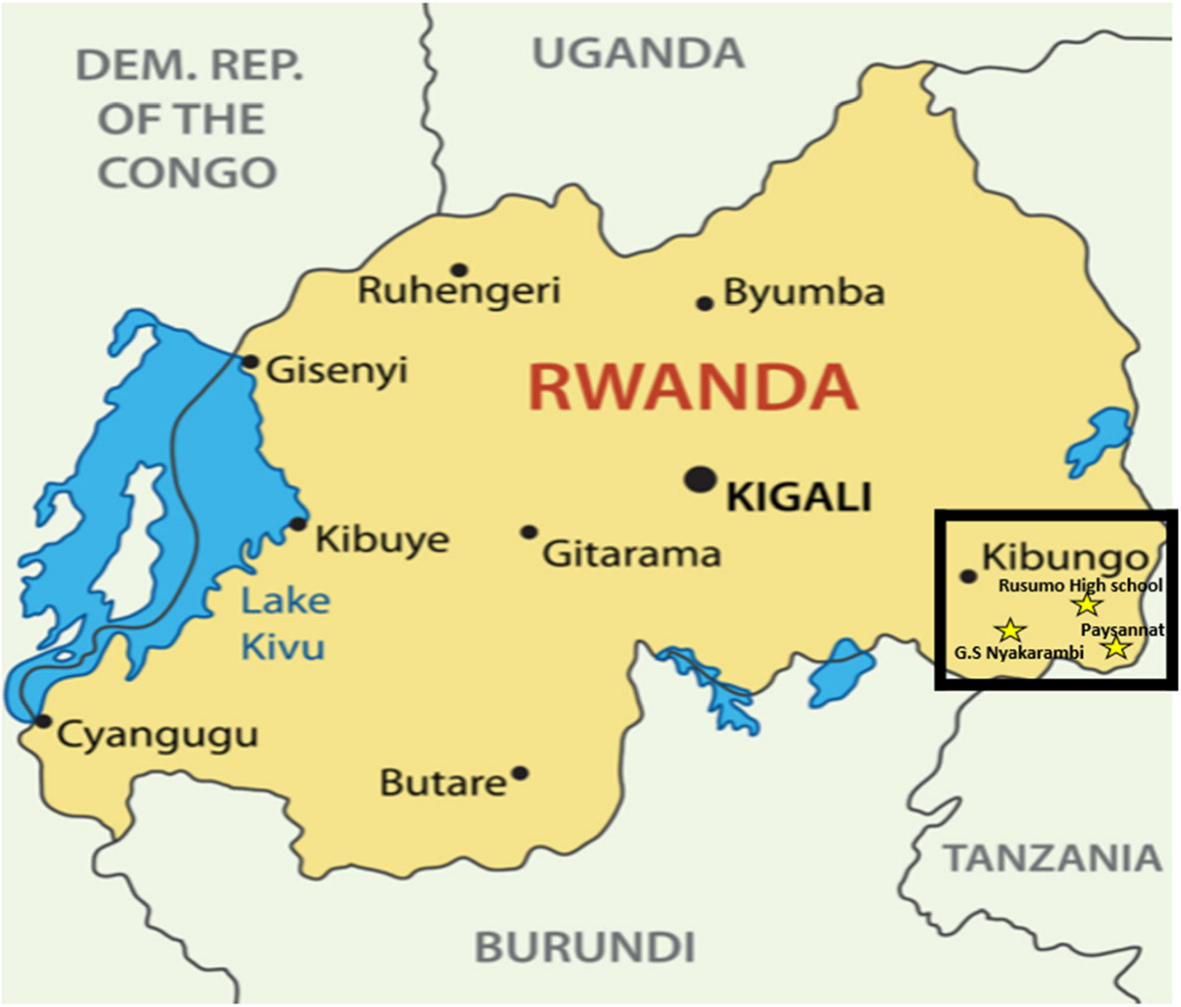
Map of participating schools in Kirehe district. Source: pavalena/Shutterstock.com

The current research project is a combination of quantitative and qualitative research, supplemented with input from local stakeholders and experts. This research project will be done within a multicenter setting including three secondary schools in Kirehe district: 1) GS Nyakarambi, 2) Rusumo High School, and 3) Paysanat Ld (Fig. 1). These three schools are included because of their geographical distribution in Kirehe district and because of their heterogeneous composition. Schools commit themselves in helping to provide students for the quantitative and qualitative parts of the study.

The study is approved by the ethical committee of the executive center, University of Rwanda, college of Medicine and Health Sciences, Institutional Review Board, approval No 158/CMHS IRB/2019. All students included in the quantitative and/or qualitative study give written informed consent themselves (18 years or older) or through their parents (younger than 18 years).

### Study population

The study includes in-school students aged 15 to 19 years old, both male and female. Translated to the Rwanda school system, this refers to students from secondary 3 to 6. No other specific in- or exclusion criteria are formulated, not regarding sexual activity nor regarding adolescent pregnancy.

### Study phases and data collection

The duration of the project is 3 years and is from January 2019 until December 2021. The first year is used for preparatory research as fundament for the development of de PEP, including 1) a local network event to obtain input from important local stakeholders and experts, 2) quantitative research to examine adolescents’ level of knowledge and attitudes regarding SRH education, and 3) qualitative research to explore adolescents’ attitudes and needs regarding SRH more in depth. At the end of the first project year and during the second project year, the framework and accompanying script of the PEP will be developed, followed by the actual implementation and evaluation in the third project year.

#### Input from local stakeholders and experts

Taking into account the social context and complexity of adolescent pregnancy and SRH, input from local stakeholders and experts is considered necessary for succeeding of the project. These events target experts and stakeholders in the field of health care and education, such as school teachers and directors, delegates of hospitals or medical centers, and delegates of local policies. Local network events are on the agenda at the start, halfway and at the end of the project.

The aim of these events is to implement the research and the developed PEP maximally within daily life of young adolescents as well as ensuring that the PEP can be implemented smoothly next to the numerous educational programs and efforts in the context of SRH. Five main topics are formulated for the events, being 1) different views on Rwandan prevention strategies for adolescents pregnancies, 2) learned lessons from previous experiences and projects, 3) strengths of and necessary conditions for the PEP, 4) expected barriers and how to tackle these, and 5) other opportunities and valorization options of the research project.

#### Quantitative study

The first study phase comprises the quantitative part in which group 3 to 6 students from the three aforementioned schools are questioned regarding SRH. For this, the Illustrated Questionnaire for Interview Surveys with Young People was selected [13]. This survey is made available by the World Health Organization (WHO) and its purpose is to serve as a point of departure when examining the SRH of teenagers or young people who have reached puberty but have not yet married or entered stable cohabiting relationships. The survey is designed to document knowledge, beliefs and behavior in the domain of SRH and should be viewed as a tool to address the needs of teenagers and young people as cornerstone for interventions. This is an important perspective as the outcome of this survey will be used as input for the development of the peer to peer (PTP) education program. The instrument is constructed in such a manner that it should always be adapted to the local context, in this case to the Rwandan context. So in a first step, the original IQISYP in English is adapted to meet the expired input for the PTP education program. Second, the adapted version is being translated in Kinyarwanda and back translated into English. Only minor changes in answer options were needed to make sure that all questions and answers are clear for use in Kinyarwanda.

The slightly adapted IQISYP is developed in collaboration with local field workers. These include earlier employees of non-governmental organizations in the field of SRH in Rwanda and lectures / researchers of the Rwandan Midwifery and Nursing programs. The final survey is made of five main sections, being 1) background characteristics, 2) sources of information on, and knowledge of, SRH, 3) Knowledge and ever-use of contraceptive methods, 4) knowledge and attitudes towards Human immunodeficiency virus (HIV)/aids and sexually transmitted diseases (STDs), and 5) use and perceptions of health services. A detailed description of these five topics is given below. Sexual ideology/attitudes to gender, condom use and attitudes, homosexual experience, and protective or risk behavior were removed as topics on such, but were processed shortly within other sections. These topics seemed less relevant in view of the PTP education program and processing or removal of items led to a shortened survey of seven pages.

The *background section* questions the respondents’ sex (male / female), age (day, month and year), class/grade (secondary 3 to 6), religion (None / catholic / protestant / Muslim / other) and importance of religion (very important / important / not important), use of alcohol (no / yes / number of days using alcohol) and tobacco (no / yes / number of cigarettes per day), sexual intercourse (no / yes), use of contraceptives (no / yes / which ones do you use / where do you by?), (earlier) pregnancy (no / yes) and children (no / yes / number of children).

Section two about *sources of information on and knowledge of reproductive health* questions the most important and the preferred source about puberty in general (i.e. the way in which boys’ and girls’ bodies change during the teenage years), of sexual and reproductive systems of men and women (i.e. where eggs and sperm are made and how pregnancy occurs), and about relationships (i.e. how boys should treat girls and vice versa). Answer options include school teacher, mother, father, brother, sister, other family members, friends, doctors, books/magazines, films/videos, other (specify), and no one. Furthermore, attendance on school classes about SRH is questioned (no / yes) and what respondents think about the number of classes (more / less / about right). Next, the survey questions if respondents have ever discussed sex-related matters with their father (often / occasionally / never) or mother (often / occasionally / never). Final, four statements covering reproductive health issues are listed with answer options “true”, “false” and “don’t know/not sure”. The statements include “a girl/woman can get pregnant on the very first time that she has sexual intercourse”, “a girl/woman stops growing after she had sexual intercourse”, “masturbation causes serious damage to health” and “a girl/woman is most likely to get pregnancy if she has sexual intercourse half way between her periods”.

The third section focusses on the *knowledge and use of contraceptive methods*. Six contraceptive methods are listed (pill, injection, condom, emergency contraceptive pills, withdrawal and periodic abstinence) with a short explanation (e.g. condom – a man can put a rubber device on his penis before intercourse). Respondents are asked if they know these methods (no / yes (spontaneous or prompted). When they indicate “yes”, they are asked if they know any place or person where young people could obtain this method (no / yes). In addition, it is questioned if respondents have heard of other methods. They can circle the following options: IUD, implant, jelly/foam, female sterilization, male sterilization and other (specify). Hereafter, respondents circle the methods they think are most suitable for young people and the methods they or their sexual partner have ever used in case of experienced sexual intercourse. Answers that can be circled are pill, injection, condom, emergency pill, withdrawal, periodic abstinence, other (specify).

The fourth section relates to the *knowledge and attitudes towards HIV/Aids and STDs*. Respondents’ knowledge is examined by questioning whether they have heard of HIV/aids (no / yes) or other STDs (no / yes), and what kind of symptoms they are known when a men (discharge from penis / pain during urination / ulcers or sores in genital area / other (specify)) or a woman (vaginal discharge / pain during urination / ulcers or sores in genital area / other (specify) is infected. The occurrence of STDs (once / more than once / never) and whether respondents’ or their partner have ever used a condom (no / yes) is questioned. Next, some statements about HIV/aids are given and respondents should rate “true”, “false” or “don’t know”. Statements are “it is possible to cure aids”, a person with HIV always looks emaciated or unhealthy in some way” and “people can take a simple test to find out whether they have HIV”. Finally, twelve opinions are listed about condoms and respondents are questioned whether they agree, disagree or don’t know. For example, an opinion to judge is “a girl can suggest to her boyfriend that he uses a condom” or “condoms reduce sexual pleasure”.

The final section includes the *use and perceptions of health services*. Respondents must indicate if they have ever visited a health facility or doctor of any kind to receive services or information on contraception, pregnancy, abortion or STDs (no / yes). In addition, it was questioned whether respondents have seen any poster on contraception, given brochures on contraception, attended a talk on contraception, requested contraceptive services during the consultation, felt comfortable enough to ask questions and whether they felt questions were answered adequately at this facility (no / yes).

This survey is taken at the beginning and the end of the project. It is printed on paper and will be divided at each class room. Researchers will stay present and available at each class room to maximize respondents’ input and comfort in case of questions.

#### Qualitative research

In combination with the survey at the beginning of the project (T1), six focus group interviews are organized with Kirehe in-school students to explore their SRH knowledge and attitudes more in depth and to better understand their needs and the possible role of a PEP program in the prevention of adolescent pregnancies. It is opted that focus groups either consist of girls or boys per age category, which are 15 years (Rusumo High School), subsequently 16 to 17 years (Paysannat L.) and finally 18 to 19 years old (G.S. Nyakarambi). Written informed consent will be obtained from parents of adolescents aged 15 to 17 years old, while informed consent will be obtained from 18 to 19 year old students themselves.

Focus group interviews are led by a UR lectures assisted by a Belgian master student in Nursing. Interviews will be held in classrooms, located quiet and safe in the school area, and foreseen of a table with chairs. The interviews will be mainly done in Kinyarwanda. A native speaking Rwandan lecturer is pointed out as first interviewer, while the Belgian student acts mainly as observer to take field notes of nonverbal communication and as back-up for substantive support. A semi-structured topic list is drawn up from the literature and matched with the main scope of the project. After a general introduction by the interviewer, name tags with letters A to H are given to participants in order to anonymize the obtained data as much as possible.

#### Development and pre-testing of the PEP

The development of the actual PEP, consisting of a manual and two trainer modules, starts after retrieving insights from the preparatory quantitative and qualitative studies. The overall aim is to ‘empower’ Kirehe adolescents through the PEP. The Health Promotion Glossory of the World Health Organization (WHO, 1998) defines ‘empowerment’ as ‘gain more control over decisions and actions that affect people’s lives in general’. In an attempt to succeed in the empowerment of Kirehe adolescents in the context of SRH, it is of importance to increase the level of SRH knowledge and skills, since these are both necessary conditions for empowerment [14].

Peer education is the process whereby trained facilitators assist a subset of the target population (= trained peers) to ‘educate their peers in an adequate and structured manner, recognize individuals in need of additional help and refer them for assistance, and advocate for resources and services for themselves and their peers [15].

To ensure that the developed PEP is effective, the Youth Peer Education Toolkit will be used as theoretical framework. This toolkit was developed to build the capacity of local non-governmental organisations to design, implement, supervise, monitor, and evaluate effective SRH PEPs.

When the framework of the program is made, it is necessary that healthcare workers are sufficiently equipped with the necessary knowledge and skills to accurately facilitate peer exchange in the context of SRH. It is opted to focus on future health care workers (last year Midwifery and Nursing students) as they are already involved in SRH education from their learning programs – but in a lesser extent than pursued - so they need minimum substantive training. Furthermore, these students are more closely related to the daily life of adolescents, since they are often still young adults. And finally – and perhaps most importantly – by intervening in health care workers in training, we enhance the probability of evolving to a more sustainable and open professional climate regarding SRH. To date, it is demonstrated that SRH education is seen as “part of the job” and very uncomfortable for many professionals instead of a common prior health theme.

**Fig. 2:**
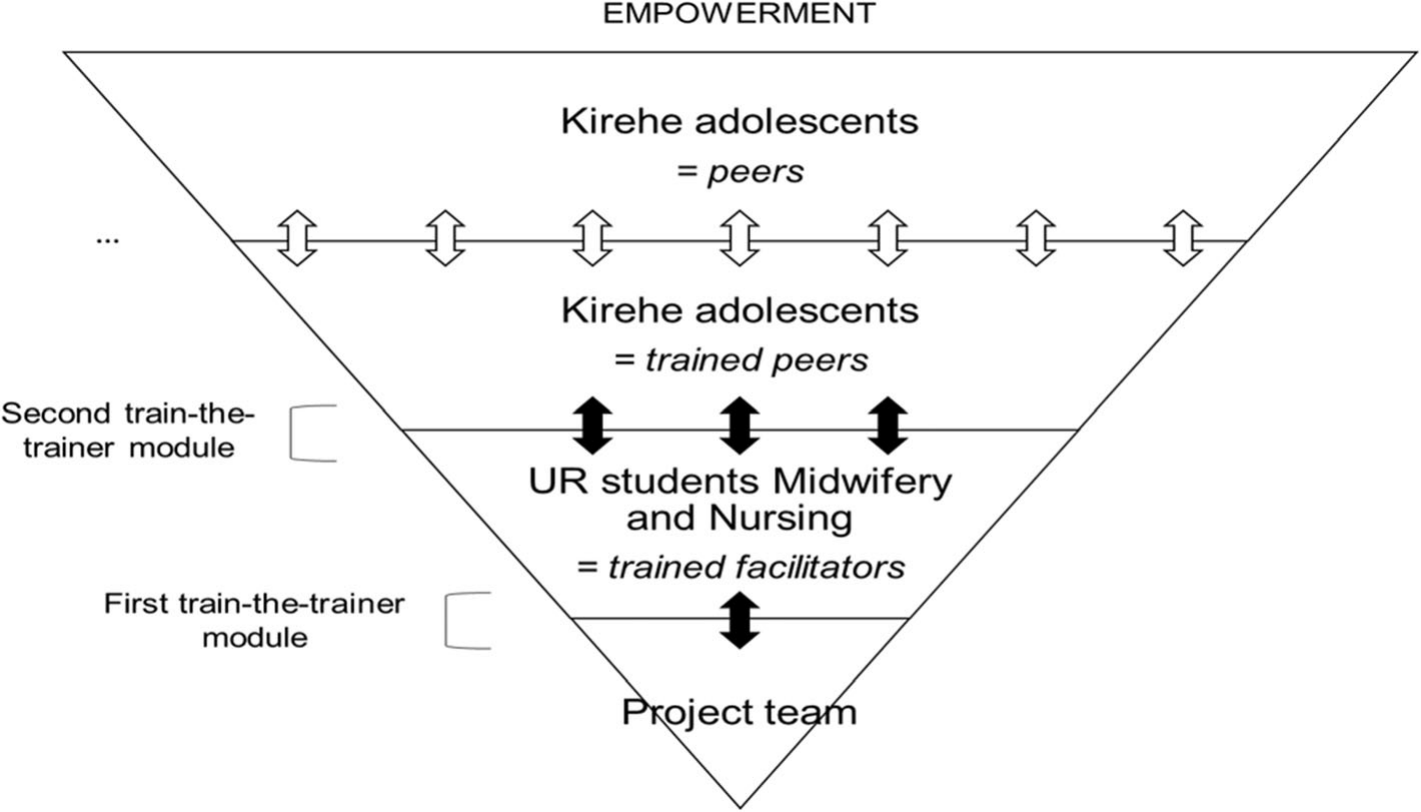
Concept of the peer education program. Source: own design

This research project intends to insert SRH education and peer training as a module into the UR Midwifery curricula (= first train-the-trainer module where UR lectures or researchers train UR students Midwifery) (Fig. 2). Next, the project steps forward to the peer education aspect. A selected group of Midwifery students (= trained facilitators, *N* = 6) will be instructed within their practical internship in the first term to enroll the second train-the-trainer module in which they train a selected group of Kirehe students (= trained peers) under the supervision of the project team. A pool of 30 peers is aimed at, of an equal number of voluntary girls and boys, and each of the three schools delivering ten students of different ages between 15 and 19 years old. In order to maximally attract and motivate adolescents to become a peer, and to watch over the feasibility of the project, we will organize together with students Midwifery three times a two-day training in the first trimester on the most relevant SRH topics and communication skills. Next to the training of peers, UR students Midwifery will be involved in peer selection and recruitment. Trained UR students Midwifery will be instructed to apply their knowledge and skills to class- and schoolmates.

After a period of approximately 6 months following the second train-the-trainer module, focus group interviews with the trained peers will be executed together with students Midwifery. We want to understand what barriers and opportunities emanate from the program, so that we can optimally fit the program to the local context. Furthermore, to evaluate whether the project resulted in more empowered adolescents, a post-measurement of the adolescents’ level of knowledge, skills and attitudes towards SRH will be organized after the third phase of the train-the-trained module for peers. These deliverables and best practices will be communicated to the project’s stakeholders. In this manner, built-up partnerships can be nourished and maintained, resulting into a more sustainable prevention strategy in the battle against adolescent pregnancies.

### Data analyses

The mixed study design allows different types of data analyses; several analytical procedures will be considered for the quantitative part, while more in-depth analyses are foreseen for the qualitative part.

Analytical procedures will include descriptive and cohort study analyses, performed in SPSS software. A specific *P*-value of significance will be determined based on the number of tests that will be performed for a specific research question. The normality of continuous variables will be checked with normality tests (Kolmogorov-Smirnov or Shapiro Wilk’s test). Depending on the normality outcomes, parametric (Student-t, one-way AN(C)OVA, pre and post-test measures, Chi2, Pearson, multiple and logistic regression test) or non-parametric (Mann–Whitney U, Kruskall Wallis, Spearman and Friedman tests) tests will be used.

Interviews will be audio-recorded and saved safely by the researchers. Following at-verbatim transcription, recorded interviews will be deleted from the dictaphones. Transcriptions will be coded in NVivo 12 according to the principles of thematic analysis. The obtained data will be triangulated by peer debriefing and main findings will be schematically conceptualized for increasing transferability.

### Power calculation

No power calculation has been performed for the quantitative study. The three participating schools are chosen with respect to the practical feasibility of the project. Besides, the choice for these three schools in the three most challenging regions in Kirehe district is based on the number of pregnancies in in-school adolescents (see introduction). However, in comparison with similar studies, the number of included adolescents seems acceptable in order to obtain reliable data outcomes.

### Start of the study and expected results

This project started in January 2019. In the first project year and the first half of the second project year, a network event took place, six focus group interviews were done, the baseline measurement was taken and the PEP program has been developed. First research papers on these data are expected to be published by the end of 2020 and in 2021. Research data from the PEP program, the pre versus post-measurement and the focus group interview with adolescents being pregnant or having one or more children will probably be published in the second half of 2021 and 2022.

## Discussion

This paper presents the emergence and development of a school-based PEP uncovering and tackling important determinants in the prevention of adolescent pregnancies in Kirehe district, Rwanda. Unique in this project is that future health care workers are being equipped with the necessary knowledge and skills in order to play a prominent role in this Rwandan health care challenge. By strengthening the Midwifery curricula in health care promotion, this research project is believed to contribute to a more profound social change since specific SRH-related skills and knowledge become a more common and structural part in the Kirehe – and broader Rwandan – health care. The project is designed so that students Midwifery can work on SRH outreaching activities as part of the curriculum and embedded in practical internships and theses. Updating and embedding topics of SRH in the existing curricula guarantees the sustainability of the project.

In order to reassure a continuation of the peer education aspect in Rwandan secondary schools, additional efforts will be made to encourage in-school adolescents to seek information by trained peers. Teachers and school administrators are being involved in the network events and individually approached to encourage and motivate students in their school to talk to their peers. In addition, actions will be undertaken by the research team and teachers and school administrators to make peers sufficiently visible in the school environment (e.g. list with trained peers at school entrance or other accessible and good visible places at school).

The greatest challenge in this research project is to include the more modest and perhaps most vulnerable in-school adolescents. In an attempt to avoid this bias, close collaborations are set up with school teachers and directors through the network events and as part of a more structural partnership between the UR and the participating schools. These local stakeholders are involved in the peer selection process and the enrolment of the actual PEP. In addition, other important community members such as delegates from local political and religious parties will be present at the events organized within this research project.

The focus on in-school adolescents might also feed the concern that a prominent part of the at-risk population for adolescent pregnancy will be missed, since it is well known that adolescent pregnancy is predominantly seen in lower class households [16]. However, particularly for the Kirehe district, the opposite counts. In addition, it can be postulated that an in-school prevention strategy seeps through into the wider community as trained peers may use their gained knowledge and skills outside the school context. Notwithstanding, mixed strategies targeting the school as well as the community would be most optimal. An effective mixed prevention strategy can be developed by integrating the gained experience and knowhow from current and previous research, and can become subject of a next Vlir-uos research proposal.

Next to the development of a PEP, a side path of the project is to conduct a focus group interview with adolescent pregnant girls and adolescent mothers. This will certainly be a strong supplement to the main study and provide the researchers with a better understanding of the underlying mechanisms specific to the phenomena of adolescent pregnancy.

To conclude, it is believed that the current Vlir-uos project has strong potential to contribute in a significant manner to more empowered Kirehe adolescents who adopt safer sexual behaviors. Further investment in mixed prevention strategies remains undisputable and a necessary condition for further improvement of adolescents’ sexual health in Rwanda.

## Data Availability

The data that support the findings of this study are available from VLIR-UOS but restrictions apply to the availability of these data, which were used under license for the current study, and so are not publicly available. Data are however available from the authors upon reasonable request and with permission of VLIR-UOS.

## Abbreviations

CLADHO: Collectif des Ligues et Associations de Défense des Droits de l’Homme au Rwanda
e.g.: Example given
IQISYP: Illustrated Questionnaire for Interview Surveys with Young People
PEP: Peer education program
SRH: Sexual and reproductive health
STDs: Sexual transmitted diseases
UCLL: University Colleges Leuven-Limburg
UNFPA: United Nations Population Fund
UR: University of Rwanda
WHO: World Health Organization
